# Sinulariolide Suppresses Human Hepatocellular Carcinoma Cell Migration and Invasion by Inhibiting Matrix Metalloproteinase-2/-9 through MAPKs and PI3K/Akt Signaling Pathways

**DOI:** 10.3390/ijms160716469

**Published:** 2015-07-20

**Authors:** Yu-Jen Wu, Choo-Aun Neoh, Chia-Yu Tsao, Jui-Hsin Su, Hsing-Hui Li

**Affiliations:** 1 Department of Beauty Science, Meiho University, Pingtung 91202, Taiwan; E-Mail: x00002180@meiho.edu.tw; 2 Department of Research, Pingtung Christian Hospital, Pingtung 90059, Taiwan; E-Mail: neohca@gmail.com; 3 Graduate Institute of Marine Biotechnology, National Dong Hwa University, Pingtung 94450, Taiwan; E-Mails: tsao-chiayu@umail.hinet.net (C.-Y.T.); x2219@nmmba.gov.tw (J.-H.S.); 4 Industry Academia Collaboration Center, National Museum of Marine Biology and Aquarium, Pingtung 94450, Taiwan

**Keywords:** hepatocellular carcinoma (HCC), *Sinularia flexibilis*, sinulariolide, matrix metalloproteinase-2/-9, migration, invasion

## Abstract

Sinulariolide is an active compound isolated from the cultured soft coral *Sinularia flexibilis*. In this study, we investigate the migration and invasion effects of sinulariolide in hepatocellular carcinoma cell HA22T. Sinulariolide inhibited the migration and invasion effects of hepatocellular carcinoma cells in a concentration-dependent manner. The results of zymography assay showed that sinulariolide suppressed the activities of matrix metalloproteinase (MMP)-2 and MMP-9. Moreover, protein levels of MMP-2, MMP-9, and urokinase-type plasminogen activator (uPA) were reduced by sinulariolide in a concentration-dependent manner. Sinulariolide also exerted an inhibitory effect on phosphorylation of c-Jun N-terminal kinase (JNK), extracellular signal-regulated kinases (ERK), phosphatidylinositol 3-kinase (PI3K), Akt, Focal adhesion kinase (FAK), growth factor receptor-bound protein 2 (GRB2). Taken together, these results demonstrated that sinulariolide could inhibit hepatocellular carcinoma cell migration and invasion and alter HA22T cell metastasis by reduction of MMP-2, MMP-9, and uPA expression through the suppression of MAPKs, PI3K/Akt, and the FAK/GRB2 signaling pathway. These findings suggest that sinulariolide merits further evaluation as a chemotherapeutic agent for human hepatocellular carcinoma.

## 1. Introduction

Hepatocellular carcinoma (HCC) is a highly lethal cancer, ranks as the fifth leading cause of cancer death worldwide, which receives much public health attention [1]. HCC presents as an aggressive tumor type and the median survival after diagnosis is only 6 to 20 months [2,3]. More than half a million new cases are discovered each year, and the incidence in patients with cirrhosis ranges from 2.5%–7% [4]. Standard treatment options of HCC include surgical resection, liver transplantation, transarterial chemoembolization (TACE), percutaneous ethanol injection (PEI), radiofrequency ablation (RF), and target therapy, depending on cancer stage and suitable candidates [5]. The side effects of chemotherapy agents remain a major concern of these aggressive treatment options. Therefore, the potential agents for the treatment of hepatocellular carcinoma agents with fewer side effects are urgently needed in clinicals. Accordingly, therapy strategies focus on inhibiting growth of existing tumors, and blocking cancer cell invasion and metastasis.

Malignant tumor progression depends upon the ability of invasion and metastasis. Invasion and metastasis are principal characteristics of malignancy with poor clinical outcomes. Matrix metalloproteinase (MMP)-2 and -9 are proteolytic enzymes that are highly expressed in hepatocellular carcinoma [6]. These enzymes function to degrade the environmental extracellular matrix (ECM) and the basement membrane. The activity of MMPs is inhibited by endogenous tissue inhibitor of metalloproteinases (TIMPs), which are specific inhibitors of MMPs, and the imbalance between MMPs and TIMPs contribute to the degradation or deposition of the ECM. MMPs and TIMPs play an important role in hepatocellular carcinoma invasion by degrading extracellular matrix proteins [7]. Mitogen-activated protein kinases (MAPKs) play an important regulatory role in cell growth, differentiation, apoptosis, and metastasis [8]. In addition, the phosphatidylinositol-3-kinase (PI3K)/Akt signal transduction pathway is involved in the development, progression, and metastasis of various tumors [9,10,11,12].

The therapeutic applications of natural products isolated from marine soft corals have been widely investigated [13,14,15,16]. Several compounds such as diterpenes, diterpenoids, and prostanoids have been isolated from soft corals. Despite their unknown mechanisms, these compounds have been reported to exhibit the anti-cancer effects through the induction of apoptosis and cytotoxic against different cancer cell lines, such as prostate, breast, colon, melanoma, liver, oral cancer and cervical cell lines [17,18,19,20,21,22,23,24,25,26,27,28]. Sinulariolide, an active compound isolated from the cultured soft coral *Sinularia flexibilis* [15] has various biological properties, including anti-microbial and anti-cancer activities, particularly in bladder cancer, hepatocellular carcinoma, and melanoma [22,29,30]. In the current research, we will evaluate the molecular mechanism inhibiting human HCC by sinulariolide. Overall, these results could provide valuable information for drug development or potential strategies against human HCC.

## 2. Results

### 2.1. Sinulariolide Inhibited Migration and Invasion of HA22T Cells

**Figure 1:**
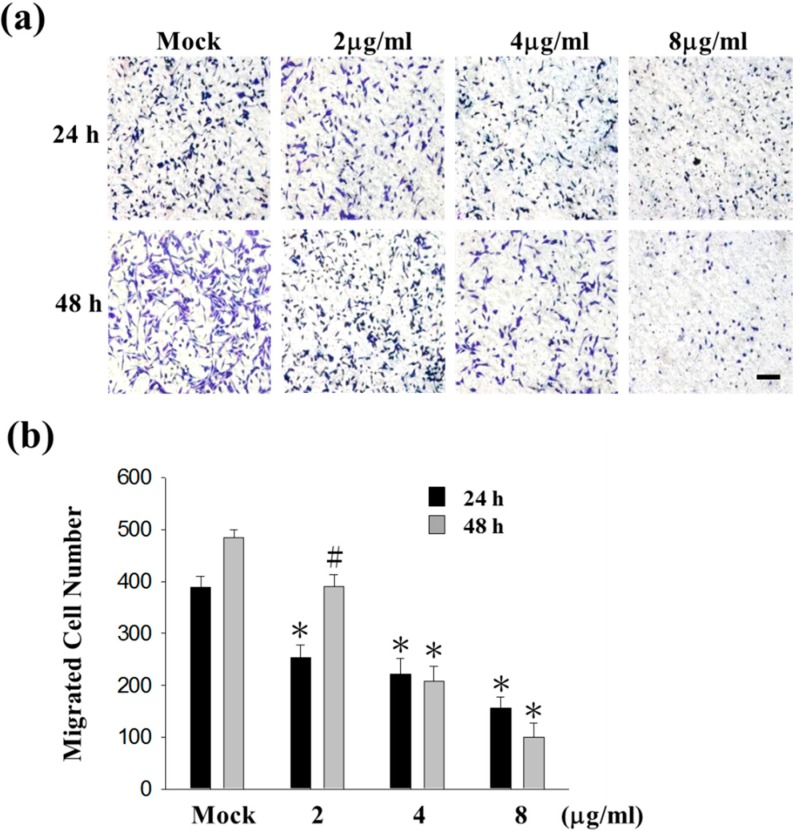

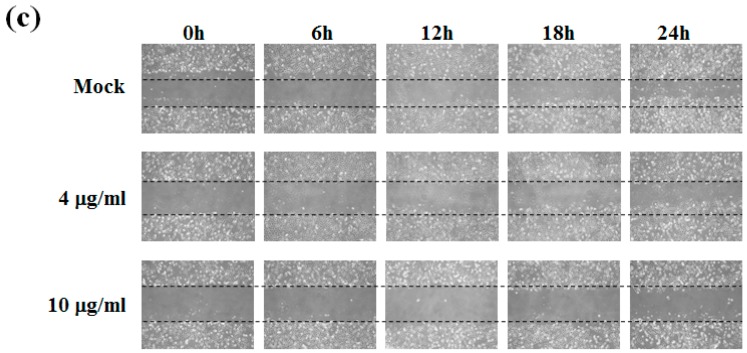
The anti-migratory effects of sinulariolide in HA22T cells. (**a**) After 24 and 48 h treating with sinulariolide, migrated HA22T cells were clearly reduced compared to control cells at the 100× magnification vision. The results shown are representative of three independent experiments. Scale bars = 20 μm. Mock: control, the DMSO-treated cell; (**b**) Quantitative analysis of figure (**a**) showed that sinulariolide dose-dependently suppresses HA22T cell migration (# *p* < 0.05, * *p* < 0.001 compared with the control); (**c**) Representative images of monolayer scratch assays supported that sinulariolide inhibited the migratory potential of HA22T cells.

**Figure 2:**
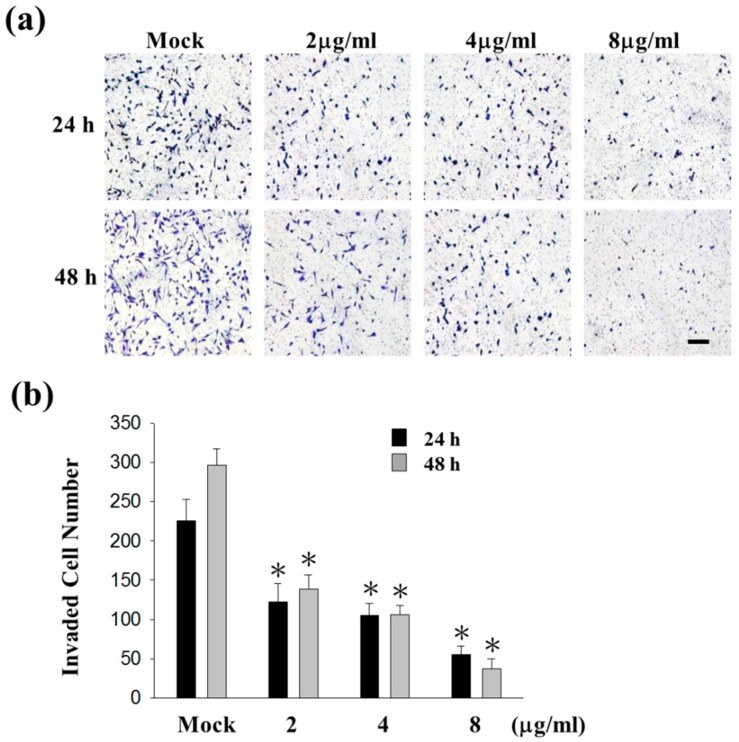
The anti-invasive effects of sinulariolide in HA22T cells. (**a**) After 24 and 48 h treating with sinulariolide, invaded HA22T cells were clearly reduced compared to control cells at the 100× magnification vision. The results shown are representative of three independent experiments. Scale bars = 20 μm. Mock: control, the DMSO-treated cell; (**b**) Quantitative analysis of figure (**a**) showed that sinulariolide dose-dependently suppresses HA22T cell invasion (* *p* < 0.001 compared with the control).

Cell matrix interaction and cell motility are important for cancer cell metastasis. To examine the potential anti-metastasis effects of sinulariolide, migration and invasion assays were performed in HA22T cells. The results indicated that HA22T cells were inhibited by sinulariolide in a concentration-dependent manner. Moreover, HA22T cells treated with 8 μg/mL sinulariolide for 24 and 48 h, the migratory abilities were reduced by 50% and 78% (Figure 1a,b), and invasive abilities were reduced by 80% and 84%, respectively (Figure 2). Monolayer scratch assays also supported that sinulariolide inhibited the migratory potential of HA22T cells (Figure 1c). These results suggest that sinulariolide is an inhibitor of hepatoma cells in migration and invasion.

### 2.2. Sinulariolide Reduced the Matrix Metalloproteinase (MMP)-2/-9 Activities of HA22T Cells

**Figure 3:**
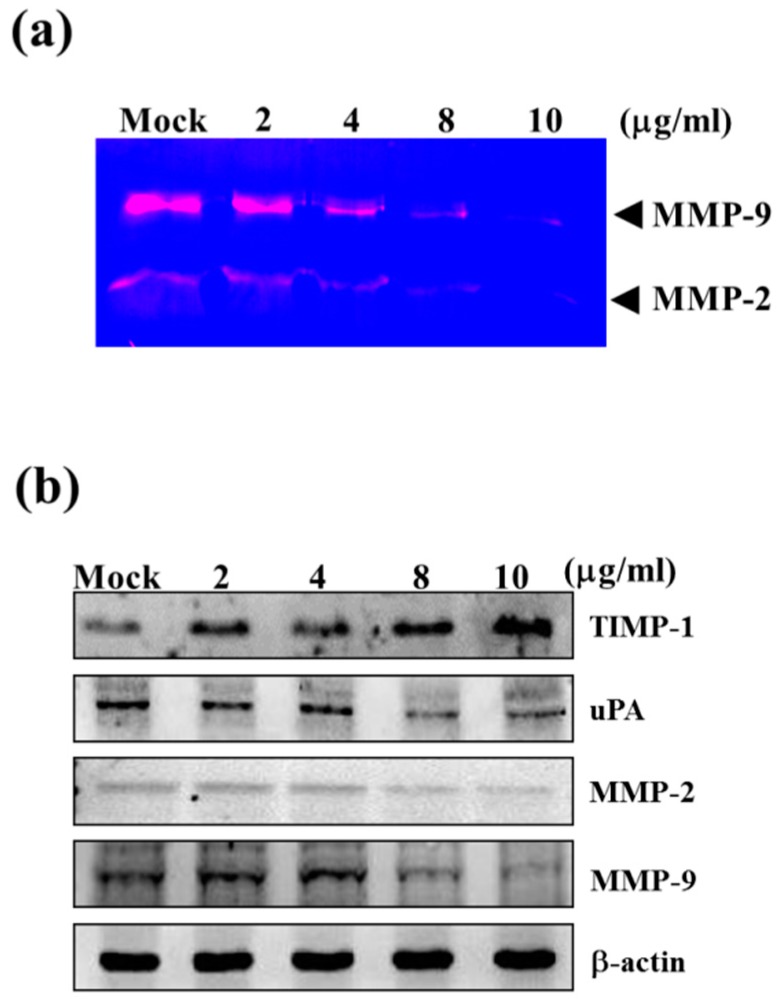
Effects of the MMP-2/-9 activities and protein levels in HA22T cells by sinulariolide. The HA22T cells were treated with different concentration of sinulariolide (0, 2, 4, 6, 8 μg/mL) for 24 h, and conditioned media and cell lysates were collected respectively for gelatin zymography assay and western blotting. (**a**) The gelatin zymography assay showed MMP-2/-9 activities were inhibited by sinulariolide in HA22T cells; (**b**) The MMP-2/-9 related proteins were validated, including TIMP-1, uPA, MMP-2, and MMP-9. The protein levels of MMP-2/-9 and uPA were decreased, but TIMP-1 was increased in HA22T cells. β-Actin was used as the internal control. Mock: control, the DMSO-treated cell.

To clarify whether the activity of MMP-2 and MMP-9 are involved in the invasion of HA22T cells, gelatin zymography assay was performed. The HA22T cells were incubated in serum-free media with sinulariolide (0, 2, 4, 6, 8 μg/mL) for 24 h, and the conditioned media were collected to analyze activities of MMP-2 and MMP-9. The result showed that sinulariolide reduced the activities of MMP-2 and MMP-9 in a conentration-dependent manner (Figure 3a).

### 2.3. Sinulariolide Down-Regulated Protein Levels of MMP-2, MMP-9, and uPA, but Increased Protein Levels of TIMP-1

The physiological activities of MMP-2 and MMP-9 are significantly related to TIMP and the urokinase-type plasminogen activator (uPA), which was involved in the invasiveness, metastasis, and prognosis of HCC [31]. The regulation of sinulariolide on the protein levels of MMP-2, MMP-9, TIMP-1 and uPA were determined by western blotting assay. The protein levels of MMP-2, MMP-9 and uPA were decreased in HA22T cells after treating with sinulariolide for 24 h, but TIMP-1 was increased (Figure 3b). The up-regulation of TIMP-1 protein levels and down-regulation of uPA may be a possible alternative strategy for the inhibition of MMP activity, with the added benefit of anti-invasion activity. We conclude that the inhibitory effect of MMP-2 and MMP-9 in HA22T cells by sinulariolide may be through the regulating of uPA and TIMP-1.

### 2.4. Sinulariolide Inhibited MAPKs and PI3K/Akt Signaling Pathway Related Molecules

**Figure 4:**
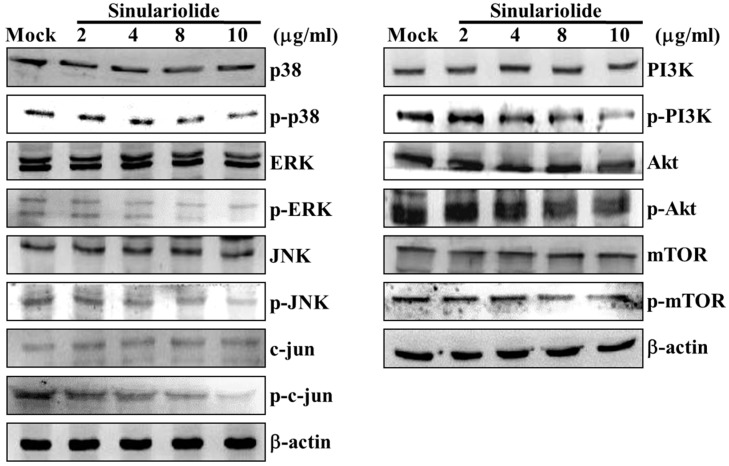
The effects of sinulariolide on MAPKs and PI3K/Akt signaling pathways. The HA22T cells were treated with different concentration of sinulariolide (0, 2, 4, 8, 10 μg/mL), and cell lysates were collected for western blotting assay. The MAPKs and PI3K/Akt related proteins were validated, including p38, p-p38, ERK, p-ERK, JNK, p-JNK, c-jun, p-c-jun, PI3K, p-PI3K, Akt, p-Akt, mTOR and p-mTOR. Sinulariolide inhibited seven phosphorylation protein levels in HA22T cells. β-Actin was used as the internal control. Mock: control, the DMSO-treated cell.

To elucidate the signaling pathways of sinulariolide in HA22T cells, the proteins involved in the MAPKs and PI3K/Akt signaling pathways were investigated by Western blotting assay, including p38, p-p38, ERK, p-ERK, JNK, p-JNK, c-jun, p-c-jun, PI3K, p-PI3K, Akt, p-Akt, mTOR and p-mTOR proteins. All the phosphorylation protein levels were decreased in HA22T cells after treating with sinulariolide, but others had no change (Figure 4). These results show that the inhibitory effect of sinulariolide may be through the MAPKs and PI3K/Akt signaling pathways.

### 2.5. Sinulariolide Inhibited GRB2 and FAK Signaling Pathways

Focal adhesion kinase (FAK) is a key mediator of signaling by integrins, a major family of cell surface receptors for extracellular matrix. FAK also plays a prominent role in tumor progression and metastasis through its regulation of both cancer cells and their microenvironments including cancer cell migration, invasion, epithelial to mesenchymal transition, and angiogenesis [32]. Growth factor receptor-bound protein 2 (GRB2) is a key molecule in intracellular signal transduction, and its signaling is critical for cell cycle progression, actin-based cell motility, epithelial morphogenesis, angiogenesis and vasculogenesis. These functions make GRB2 become a key molecules involved in spreading of solid tumors through invasion and metastasis [33].

**Figure 5:**
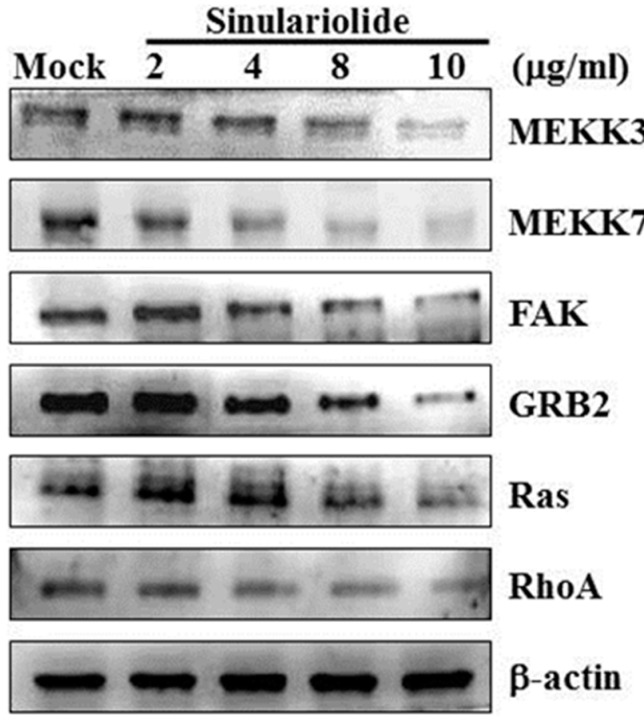
The effects of sinulariolide on GRB2 and FAK signaling pathways. The HA22T cells were treated with different concentration of sinulariolide (0, 2, 4, 8, 10 μg/mL), and cell lysates were collected for Western blotting assay. The GRB2 and FAK related proteins were validated, including MEKK3, MEKK7, FAK, GRB2, Ras, and RhoA. All the protein levels were all decreased after being treated by sinulariolide in HA22T cells. β-Actin was used as the internal control. Mock: control, the DMSO-treated cell.

To elucidate if the sinulariolide inhibit cell migration through GRB2 and FAK signaling pathway in HA22T cells, we examined the molecules that were involved in the GRB2 and FAK signaling pathway, including MEKK3, MEKK7, FAK, GRB2, Ras, and RhoA by Western blotting assay. All the protein levels were decreased in HA22T cells after treating with sinulariolide (Figure 5). These results suggest that sinulariolide suppress cell migration might through inhibiting GRB2 and FAK signaling pathways.

## 3. Discussion

### 3.1. Sinulariolide Inhibits Hepatocarcinoma Cell Metastasis and Induces Apoptosis

Metastasis is responsible for more than 90% of cancer-related mortality [34]. A critical step in tumor metastasis is the degradation of basement membrane, which is catalyzed by proteolytic enzymes, such as MMPs and TIMPs [35,36]. We found that down-regulation of MMP-2 and MMP-9 via MAPKs and PI3K/Akt signaling pathways were required in the sinulariolide-mediated invasion of hepatocarcinoma cells. In our previous report, sinulariolide exerted the cell cytotoxic on four hepatocarcinoma cell lines (Huh7, HepG2, Hep3B, and HA22T) and apoptosis-induced effects through a mitochondria-related pathway on HA22T cells [29]. In another word, sinulariolide can induced hepatocarcinoma cell apoptosis and inhibit hepatocarcinoma cell metastasis at the same time. Comprehensively, the marine compound sinulariolide has strong potential to be a pharmaceutical of human hepatocarcinoma cancer through inhibiting metastasis and induced apoptosis.

### 3.2. Sinulariolide Inhibits Multiple Signaling Pathways in HCC

FAK is overexpressed in many HCC specimens and is required for c-Met/β-catenin-driven hepatocarcinogenesis. Inhibition of FAK provides a potential strategy to treat HCC [37]. The clinical validation showed that the expression levels of both GRB2 and GAB1 proteins were significantly higher in HCC tissues than those in their adjacent non-neoplastic tissues. Moreover, the combined GRB2 and GAB1 protein expression was significantly associated with aggressive tumor progression and poor prognosis in patients with HCC. These results showed that GRB2 is a key molecule in HCC and GRB2 may be strongly related to tumor progression and prognosis in patients with HCC [38]. The uPA plays important roles with regard to HCC progression and metastasis [39]. The uPA is capable of degrading cancer tissue and the surrounding ECM and also modulate several biological processes including: cell adhesion, migration, proliferation, and gene expression under various physiological and pathological conditions. Previous studies have also demonstrated that uPA is up-regulated in human tumoral liver tissue. High level of uPA expression is related to reduce patient survival and can be considered a poor prognosis marker for HCC patients [40,41].

The three molecules (FAK, GRB2, and uPA) and related singling pathways play important role in regulating metastasis of HCC. Sinulariolide, an active compound isolated from the cultured soft coral *Sinularia flexibilis* had strong potential to be a therapeutic drug for HCC through regulating the three molecules and related singling pathways.

### 3.3. Sinulariolide Inhibits Hepatocarcinoma Cell Metastasis through Multiple Signaling Pathways

Figure 1 and Figure 2 show that 2 and 4 μg/mL of sinulariolide inhibit approximately 50% cell migration and invasion of HA22T cells, but the metastasis pathway related molecules such as p-p38, p-ERK, p-JNK, p-c-jun, p-PI3K, MMP-2, MMP-9, MEKK3, MEKK7, FAK, and GRB2 (Figure 3, Figure 4 and Figure 5) show obvious inhibition by sinulariolide at the dose of 8 and 10 μg/mL. It suggested that sinulariolide inhibits hepatocarcinoma cell migration and invasion through multiple signaling pathways, not only MAPKs and PI3K/Akt signaling pathways. The overall results infer that sinulariolide inhibits cell metastasis and induces cell apoptosis, and the signaling pathways are complicated and need more work to resolve them.

### 3.4. Both Sinulariolide and 11-epi-Sinulariolide Acetate Inhibits Hepatocellular Carcinoma

Our previous paper showed that 11-epi-sinulariolide acetate reduced cell migration and invasion in human hepatocellular carcinoma [42]. Eleven-epi-sinulariolide acetate and sinulariolide both isolated from soft coral *Sinularia flexibilis*, and they have a similar backbone. The substitute group in C-11 is hydroxyl group in sinulariolide, but 11-epi-sinulariolide acetate was determined to be 11-epi-acetyl derivative of sinulariolide. In addition, 5.32 μM 11-epi-sinulariolide acetate inhibits about 40% cell migration in HA22T [42], and 4 μg/mL (11.97 μM) sinulariolide have a similar effect (Figure 1b). It means that 11-epi-sinulariolide acetate had stronger inhibitory effects in HA22T. However, the yield of sinulariolide is much higher than 11-epi-sinulariolide acetate. One kilogram (wet weight) of *Sinularia flexibilis* can extract 3–5 g sinulariolide, but only 30–50 mg of 11-epi-sinulariolide acetate. Considering the commercial drug exploration, sinulariolide has a higher economic value than 11-epi-sinulariolide acetate. Sinulariolide has a higher potential as a therapeutic drug for human hepatocellular carcinoma.

## 4. Experimental Section

### 4.1. Materials and Chemical Reagents

Rabbit anti-human mitogen-activated focal adhesion kinase (FAK), protein kinase kinase3 (MKK3), MAPK kinase kinasekinase4 (MEKK4), Rho A, growth factor receptor-bound protein 2 (GRB2), mTOR and p-mTOR antibodies were obtained from Epitomics (Burlingame, CA, USA). Rabbit anti-human TIMP-1, Akt and p-Akt antibodies were obtained from ProteinTech Group (Chicago, IL, USA). Rabbit anti-human uPA, matrix metalloproteinase-2 (MMP-2), matrix metalloproteinase-9 (MMP-9), extracellular signal regulated kinases (ERK), phosphorylated extracellular signal regulated kinases (p-ERK), c-jun N-terminal kinase (JNK), p-JNK, c-jun, p-c-jun, p38, p-p38, phosphoinositide 3-kinases (PI3K) and phosphorylated-phosphoinositide 3-kinases (p-PI3K) antibodies were obtained from cell signaling technology (Danvers, MA, USA). Dimethyl sulfoxide (DMSO), protease inhibitor cocktail, and rabbit anti-human β-actin antibodies were obtained from Sigma (St. Louis, MO, USA). PVDF (polyvinylidene difluoride) membranes and goat anti-rabbit and horseradish peroxidase conjugated IgG were obtained from Millipore (Bellerica, MA, USA). Chemiliminescent HRP substrate was purchased from Pierce (Rockford, IL, USA). Sinulariolide was isolated from the cultured soft coral *Sinularia flexibilis*, following the protocol by Hsieh *et al.* [15], and dissolved in DMSO.

### 4.2. Cell Culture

HA22T cells were purchased from Food Industry Research and Development Institute (Hsinchu, Taiwan) and were cultured in (Dulbecco’s Modified Eagle’s medium) DMEM (Biowest, Nuaillé, France) containing 4 mM l-glutamine, 1 mM sodium pyruvate, 100 μg/mL streptomycin, 100 U/mL penicillin and 10% (*v*/*v*) fetal bovine serum, in a 37 °C humidified atmosphere with 5% CO_2_.

### 4.3. Cell Migration Assay

Cell migration assay was assayed according to the methods described by Su *et al.* [21]. HA22T cells were seeded onto a Boyden chamber (Neuro Probe, Cabin John, MD, USA) at 10^4^ cells/well in serum-free media for 24 h. HA22T cells were than incubated with different concentration of sinulariolide (0, 2, 4, and 8 μg/mL) and were allowed to migrate for 24 h. The migrated cells on the lower site were fixed by 100% methanol and then stained with 5% Giemsa (Merck, Darmstadt, Germany). Cell numbers were observed and counted at 100× light microscope.

### 4.4. Wound Healing Assay

To determine cell motility, HA22T cells were seeded in a 12-well tissue culture plate. After one day, the center of the cell monolayers was scraped with a sterile micropipette tip to create a straight zone (gap) of constant width. Then, each well was washed with Phosphate-buffered saline (PBS), and HA22T cells were exposed to various concentrations of sinulariolide (0, 4, and 10 μg/mL). Wound closure was monitored and photographed at 0, 6, 12 and 18 h with a Nikon inverted microscope.

### 4.5. Gelatin Zymography Assay

HA22T cells (1 × 10^5^ cells/well) cultured in 12-well plates were incubated in serum-free DMEM with different concentration of sinulariolide (0, 2, 4, 8, 10 μg/mL). After 24 h, MMPs released from HA22T cells and the conditioned medium was assayed using gelatin zymography (8% zymogram gelatin gels) according to the methods reported by Huang *et al.*, with some modification [37,38]. Briefly, the culture medium was electrophoresed in a sodium dodecyl sulfate polyacrylamide gel electrophoresis (SDS-PAGE) gel containing 0.2% gelatin. The gel was then washed twice in a wash buffer (100 mM NaCl and 2.5% Triton X-100 in 50 mM Tris-HCl, pH 7.5) and subsequently transferred to a reaction buffer (200 mM NaCl, 0.02% NaN_3_, 1 µM ZnCl_2_, 1 mM CaCl_2_, 2% Triton-X 100, in 50 mM Tris-HCl, pH 7.5) for enzymatic reaction at 37 °C with shaking overnight. Finally, the gel was stained with Coomassie blue, and destained in 10% acetic acid (*v*/*v*) and 20% methanol (*v*/*v*) and quantified using Image J software (http://imagej.nih.gov/ij/).

### 4.6. Cell Invasion Assay

HA22T cells were suspended in serum-free DMEM, and added to the upper chamber of the transwell chamber coating with Matrigel. The DMEM with different concentration of sinulariolide (0, 2, 4, and 8 μg/mL) was added to the lower chamber. Cells were then incubated for 24 and 48 h and fixed in neutral formalin. The Matrigel and cells in upper chamber were removed, followed by Giemsa staining. Several fields were randomly selected under a light microscope followed by cell counting to determine the extent of invasion.

### 4.7. Western Blot Analysis

The treated samples and the control samples (25 μg) were separated by 12.5% SDS-PAGE, and then transferred onto PVDF membrane for 1.5 h at 400 mA using Transphor TE 62 (Hoeffer) and then proteins transfer checked by staining with Ponceau S solution. The membranes were incubated with 5% dehydrated skim milk to block nonspecific protein bindings, and then incubated with primary antibodies at 4 °C overnight. The primary anti-human TIMP-1, uPA, MMP-2, MMP-9, p38, p-p38, ERK, p-ERK, JNK, p-JNK, c-jun, p-c-jun, PI3K, p-PI3K, Akt, p-Akt, mTOR, p-mTOR, MEKK3, MEKK7, FAK, GRB2, Ras, RhoA, and β-actin antibodies were used. The second antibodies (horseradish peroxidase conjugate goat anti-rabbit, 1:5000 in blocking solution) were added and incubated for 2 h at 4 °C and then visualized using chemiluminesence (Pierce Biotechnology, Rockford, IL, USA).

### 4.8. Statistical Analysis

Data of cell migration assay and invasion assay were pooled from three independent experiments. The results were expressed as mean ± standard error of mean (SEM). Data acquisition and analysis of variance (ANOVA) was carried out by the Tukey-Kramer test, using GraphPad InStat 3 software (GraphPad Software, San Diego, CA, USA) to determine significant differences (*p* ≤ 0.05) compare to experimental groups.

## 5. Conclusions

**Figure 6:**
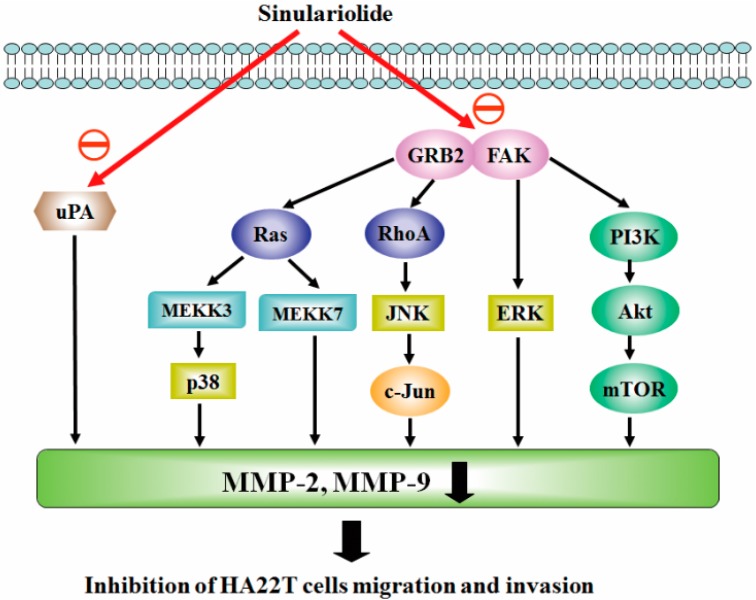
Proposed signaling pathways for sinulariolide-mediated inhibition of HA22T cell migration. The effect of sinulariolide is most likely achieved through the inhibition of FAK and GRB2, which regulates MMP-2/-9 expression through the MAPK and PI3K/Akt signaling pathways. Red arrows means sinulariolide inhibit the pathways. Black arrows means the upper molecules induced the following molecules.

In conclusion, our results have established that natural marine compound sinulariolide possesses anti-cancer activity through the suppression of cell invasion and migration in HA22T cells. Gelatin zymography and Western blotting assay indicates MMP-2/-9 and related signal pathways were regulated through the cell metastasis progress. Overall, this study indicated that sinulariolide-inhibited cell metastasis can be summarized as shown in Figure 6. Sinulariolide-inhibited cell metastasis might be related to the suppression of the MAPKs and PI3K/Akt signaling pathways. Further, *in vivo* evaluation of the anti-metastasis activity of sinulariolide in an animal model is needed. Our study provides a potential therapeutic drug in hepatocellular carcinoma.
